# The use of Enhanced Vegetation Index for assessing access to different types of green space in epidemiological studies

**DOI:** 10.1038/s41370-024-00650-5

**Published:** 2024-02-29

**Authors:** Amy Mizen, Daniel A. Thompson, Alan Watkins, Ashley Akbari, Joanne K. Garrett, Rebecca Geary, Rebecca Lovell, Ronan A. Lyons, Mark Nieuwenhuijsen, Sarah C. Parker, Francis M. Rowney, Jiao Song, Gareth Stratton, Benedict W. Wheeler, James White, Mathew P. White, Sue Williams, Sarah E. Rodgers, Richard Fry

**Affiliations:** 1 Swansea University Medical School, Swansea University, Swansea, UK; 2 European Centre for Environment and Human Health, University of Exeter Medical School, University of Exeter, Truro, UK; 3 Department of Public Health, Policy and Systems, University of Liverpool, Liverpool, UK; 4 ISGlobal, Barcelona, Spain; 5 Universitat Pompeu Fabra (UPF), Barcelona, Spain; 6 CIBER Epidemiología y Salud Pública (CIBERESP), Madrid, Spain; 7 School of Geography, Earth and Environmental Sciences, University of Plymouth, Plymouth, UK; 8 Public Health Wales, Cardiff, UK; 9 ASTEM Research Centre, Faculty of Science and Engineering, Swansea University, Swansea, UK; 10 Centre for Trials Research, School of Medicine, Cardiff University, Cardiff, UK; 11 Cognitive Science Hub, University of Vienna, Vienna, Austria; 12 Natural Resources Wales, Bangor, UK

**Keywords:** Enhanced Vegetation Index (EVI), Exposure assessment, Residential greenness, Epidemiological studies

## Abstract

**Background:**

Exposure to green space can protect against poor health through a variety of mechanisms. However, there is heterogeneity in methodological approaches to exposure assessments which makes creating effective policy recommendations challenging.

**Objective:**

Critically evaluate the use of a satellite-derived exposure metric, the Enhanced Vegetation Index (EVI), for assessing access to different types of green space in epidemiological studies.

**Methods:**

We used Landsat 5–8 (30 m resolution) to calculate average EVI for a 300 m radius surrounding 1.4 million households in Wales, UK for 2018. We calculated two additional measures using topographic vector data to represent access to green spaces within 300 m of household locations. The two topographic vector-based measures were total green space area stratified by type and average private garden size. We used linear regression models to test whether EVI could discriminate between publicly accessible and private green space and Pearson correlation to test associations between EVI and green space types.

**Results:**

Mean EVI for a 300 m radius surrounding households in Wales was 0.28 (IQR = 0.12). Total green space area and average private garden size were significantly positively associated with corresponding EVI measures (β = < 0.0001, 95% CI: 0.0000, 0.0000; β = 0.0001, 95% CI: 0.0001, 0.0001 respectively). In urban areas, as average garden size increases by 1 m^2^, EVI increases by 0.0002. Therefore, in urban areas, to see a 0.1 unit increase in EVI index score, garden size would need to increase by 500 m^2^. The very small β values represent no ‘measurable real-world’ associations. When stratified by type, we observed no strong associations between greenspace and EVI.

**Impact:**

- It is a widely implemented assumption in epidiological studies that an increase in EVI is equivalent to an increase in greenness and/or green space.
- We used linear regression models to test associations between EVI and potential sources of green reflectance at a neighbourhood level using satellite imagery from 2018.
- We compared EVI measures with a ‘gold standard’ vector-based dataset that defines publicly accessible and private green spaces.
- We found that EVI should be interpreted with care as a greater EVI score does not necessarily mean greater access to publicly available green spaces in the hyperlocal environment.

## Introduction

Exposure to green space in the home neighbourhood has been associated with positive impacts on physical and mental health outcomes; including mortality, cardiovascular disease and well-being [1–3]. Evidence suggests that various behavioural mechanisms such as viewing and spending time in the green space supports good health through being physically active, reducing stress, allowing social connectedness and time to relax [4, 5]. Factors such as socioeconomic deprivation may modify this relationship and studies have highlighted inequalities in the quality and accessibility to green space [6, 7]. However, longitudinal evidence is lacking [8] and there are no accepted frameworks for quantifying exposure to green space.

Therefore, definitions of exposure to green space are often nuanced, context‐specific, and application-dependent [9]. Past epidemiological and public health studies have assessed neighbourhood exposure to green space using many different measures. Vegetation indices based on remotely sensed data from satellite imagery [10–12] represent green vegetation and are widely used as a measure of greenness or green space [13]. Vector-based measures such as land cover maps [14–16], mapping agency data, crowdsourced data (e.g. openstreetmap) [17] and local government audits tend to be used to represent area based measures of exposure to green space such as size of nearest green space from the home location or proporation of an area-level boundary that contains green space. Survey data that record self-reported visits tend to represent exposure to green space as time spent in a green space or distance travelled to a green space [11, 18, 19]. Furthermore, different approaches to defining and managing the green space among local and national government bodies can present challenges to understanding the impact of green space on health and well-being outcomes, and for translating evidence into policy and action [20, 21].

Remote sensing is widely used for extracting information about the environment [22] through satellite sensors recording reflected and emitted radiance from the earth’s surface. This radiance is classified into different wavelength ranges and the reflective range (0.4–2.5 µm) is used to identify the presence of vegetation within a pixel. The nature of remotely sensed data means that there are numerous advantages compared to other approaches [23]. Data are: easily obtainable for large spatio-temporal ranges; open-source (e.g. 30 m from Landsat [24]); uniformly collected and therefore subject to less variability in the way data are defined; collected for municipal and administrative areas; and able to measure exposure in an objective and uniform way [25]. Conversely, the challenges of working with satellite data include spatial and temporal resolution, cloud cover, shadows cast by buildings in dense urban areas [26], and missing smaller urban green spaces such as trees and pocket parks found in urban areas [27, 28]. Furthermore, finding appropriately cloud-free satellite data at the correct time of year can be particularly challenging for northern-hemisphere climates. Satellite data processing includes adjustments and masks to mitigate some issues of cloud cover, but it remains challenging to work with at a national level and may lead to exposure misclassification [27].

Satellite-derived vegetation indices (VIs) such as Normalised Difference Vegetation Index (NDVI), Enhanced Vegetation Index (EVI), Soil-adjusted Vegetation Index (SAVI) and Leaf Area Index (LAI) have been found to be positively associated with mental and physical health outcomes and health-promoting behaviours [29–33]. However, when defining exposure to green space, VIs have been implemented as a measure of both exposure to greenness [34–36] and green spaces [37–40]; with studies interpreting a greater index score to represent greater access to green space [41, 42]. Greater VI values have been interpreted as representing greater access to green space by area because VIs are indicators of green vegetation which is inherently what makes a green space. However, VIs are a dimensionless measure of green reflectance and therefore may not be linearly associated with 2-dimensional measures of access to green space [43]. Recent studies have begun to acknowledge that there is a difference between greenness and green spaces when defining exposure to green space [11, 44, 45]. Despite this, there have been few attempts to examine the association between VIs and objective area-based measures of green space [27, 46] to contribute to understanding what changes in mean VI values mean to policy makers and planners. As studies begin to investigate muti-dimensional aspects of green space attributes e.g. type and quality to understand more specifically how green spaces support good health and wellbeing, it is important to understand how to interpret changes in VIs in relation to changes in vegetaion amount and type [47].

### Study overview

We wanted to evaluate the assumption that an increase in EVI is equivalent to an increase in accessible green space. To do this we used linear regression models to test associations between EVI and potential sources of green reflectance at a neighbourhood level using satellite imagery from 2018. We compared EVI measures with a ‘gold standard’ vector-based dataset that defines publicly accessible and private green spaces for 2018. We used EVI because it was developed to optimise the vegetation signal compared to NDVI by improving its sensitivity to high biomass regions and vegetation monitoring by reducing atmospheric noise [48, 49]. This was pertinent when considering Wales’ climate and topography.

We hypothesised that EVI would be positively associated with amount of publicly accessible and private green space by area (m^2^). For publicly accessible green spaces we predicted that the association would vary by green space type. Specifically, we defined our research questions as:

1. Is mean EVI positively associated with green space found within 300 m of households in Wales?
2. Are any associations modified by green space type?
3. Is mean EVI positively associated with private green space (i.e. average garden size) within 300 m of households in Wales?

## Methods

### Study background

The cross-sectional work reported in this paper was conducted as part of a wider longitudinal study called the Green-Blue Spaces project [45, 50, 51] where we developed a national level annual exposure variable for 1.49 million households over 11 years (2008–2019) using satellite derived EVI. Interpreting EVI scores in space and over time in relation to different types of green space was challenging and provoked us to conduct this cross-sectional evaluation to support the interpretation of the impacts of greenspace on mental health and wellbeing. We used 300 m buffers to coincide with World Health Organisation guidelines of green space access [52] and implemented a cross-sectional design for this investgation because we wanted to understand base-line associations before including more complex methodological considerations in the study design such as temporally aligning the EVI and aceess to green space data and seasonal changes in EVI values.

### Study area and study subjects

**Fig. 1:**
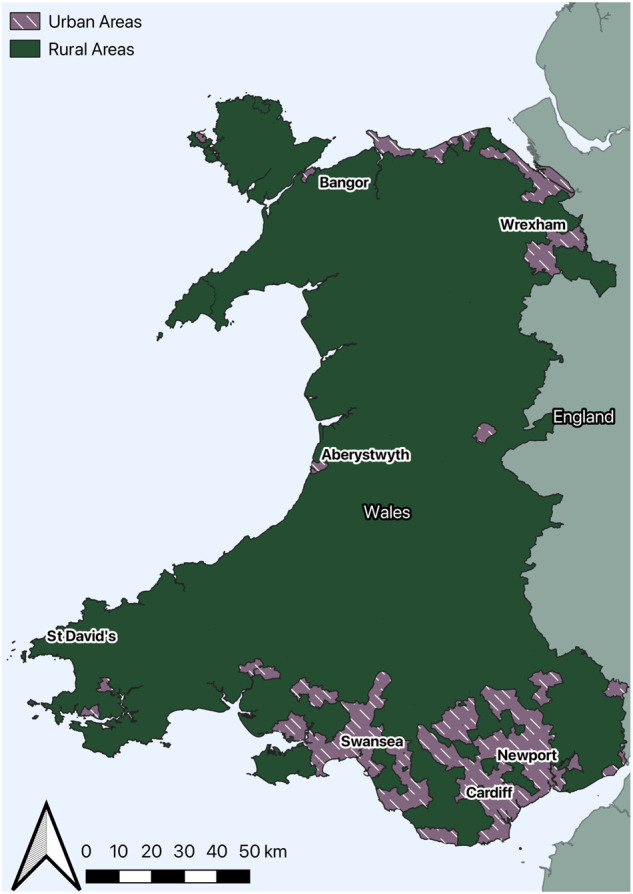
Map of Wales including urban and rural regions. The purple regions with white lines indicate urban areas in Wales. The dark green regions represent rural areas. Wales shares a boarder with England, which is represented in a light green colour.

This study was based in Wales (Fig. 1), a small nation (population: 3.17 million people; total area: 20,735 km^2^) with a moderate sea climate [53]. Wales has relatively mild winters and precipitation all year, with regions of elevated terrain and coastal exposure to prevailing westerly winds contributing to its high rainfall and cloud cover. Two thirds of the population live in cities and urban settlements.

### Generating exposure data

#### Satellite image data processing

We used Landsat 8 (30 m resolution) to create a measure of EVI for every residential address (*n* = 1.49 million) in Wales in 2018. We acquired images captured between May and July to temporally align EVI measures with peak greenness and minimise data gaps through cloud cover [54]. We pre-processed the images using the Semi-Automatic Classification Plugin tool in QGIS [55] and applied DOS1 atmospheric correction to each image [22]. We created cloud masks using the Cloud Masking for Landsat Products plugin [56] to set pixels covered by cloud in the satellite imagery to NULL. This prevented these values from influencing the final greenness metrics. We produced an annual composite image of Wales in QGIS by mosaicking different coverages together for the same year.

**Fig. 2:**
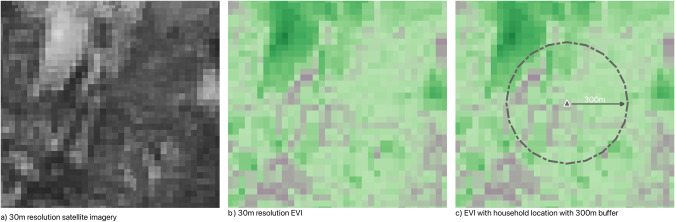
Methodological steps to calculate household level EVI. **a** represents the raw satellite data which contains 30m × 30m grid squares. **b** represents the processed satellite data. Each grid square has been assigned an EVI value. **c** shows the EVI values overlain with a 300m circular buffer around a household location.

Calculating exposure metrics: EVI was calculated using the red, blue and NIR reflectance bands found within the Landsat satellite-imagery and processed using the vegetation index GRASS tool in QGIS [57]. This resulted in a raster dataset which contained EVI values for the whole of Wales with a range of −1 (water) to +1 (vegetation) [58], with healthy vegetation values typically found in the 0.2 to 0.8 range [58]. To assign a neighbourhood EVI exposure value to each residential address location in Wales (*n* = 1.49 million), we created a Euclidean buffer of 300 m and created averages of EVI values that were found in assigned area (area of buffer with 300 m radius = 282,743 m^2^). For coastal households, the buffer was clipped to the coastline to avoid underestimates of greenness. Using this buffer layer, we performed an intersection analysis with the EVI layer to estimate the density of green vegetation (Fig. 2).

#### Topographic vector data processing

We produced a UK-relevant typology to classify green spaces for urban and rural areas (supplementary material Table A, [45]). This addressed the need for typologies that facilitate cross-disciplinary and inter-sectoral work by developing a peer-reviewed typology of green space. We consulted with more than 30 stakeholders from across Wales working in Policy, Planning and the Third Sector to iteratively develop our final typology. Farmland was not included in the typology. Although farmland constitutes large areas in rural regions, it is privately owned land and therefore not publicly accessible. We only included publicly accessible green spaces and private gardens because our overarching aim was to contribute evidence about modifiable aspects of the built environment for planning and policy guidelines. Although private gardens are not publicly accessible, we included this private green space as private garden space will make up the majority of green space within 300 m of an individual’s home location.

We used topographic vector data from multiple sources to create a map of green spaces in Wales for 2018. In summary, vector data from the UK’s national mapping agency (Ordnance Survey’s Master Map [59]) and local government audits were collated to create a dataset of all publicly accessible green spaces in Wales [60]. We categorised land parcels according to the typology (see 2.2) and extracted private garden size from OS Master Map Wales [61]. Finally, we calculated access to green space in terms of publicly accessible and private spaces within 300 m network distance of each household in Wales to coincide with World Health Organisation guidelines [52]. We defined green space access as: (1) the total area of green spaces (subset by type) and (2) average garden size, within a 300 m linear buffer of the household point location.

### Statistical analysis

We employed linear regression to investigate associations between EVI and total area of green space and average garden size (m^2^). We stratified our analyses by urban and rural settings as classified by the Office for National Statistics (ONS) urban-rural classification [62]. We also used Pearson correlation to explore the association between EVI green space by type using our typology.

## Results

Table 1 shows the three exposure assessment measures for 1.4 million households in Wales in 2018. We successfully linked 1.4 million households (95%) with an EVI and comparator vector-based green space measures. We lost comparators where garden size metrics weren’t available (*n* = 5753) or there was no access point to publicly accessible green space (*n* = 60,930), as defined by the typology, within a 300 m network distance of a household. A consort diagram of data linkage is included in the supplementary material (Fig. A).

**Table 1:** Descriptive statistics for Enhanced Vegetation Index and total green space area (m^2^) within 300 m of household locations in Wales and average garden area (m^2^).

	*Average EVI*	*Total green space area (m*^*2*^*)*	*Average garden area (m*^*2*^*)*
*Mean*	0.28	54,673	275
*Standard deviation*	0.10	37,914	331
*Minimum*	0.00	0.00	0.00
*25%*	0.22	25,300	140
*50%*	0.27	47,102	208
*75% centile*	0.34	76,601	286
*Maximum*	0.81	276,225	40,349

### National temporal and spatial variation

**Fig. 3:**
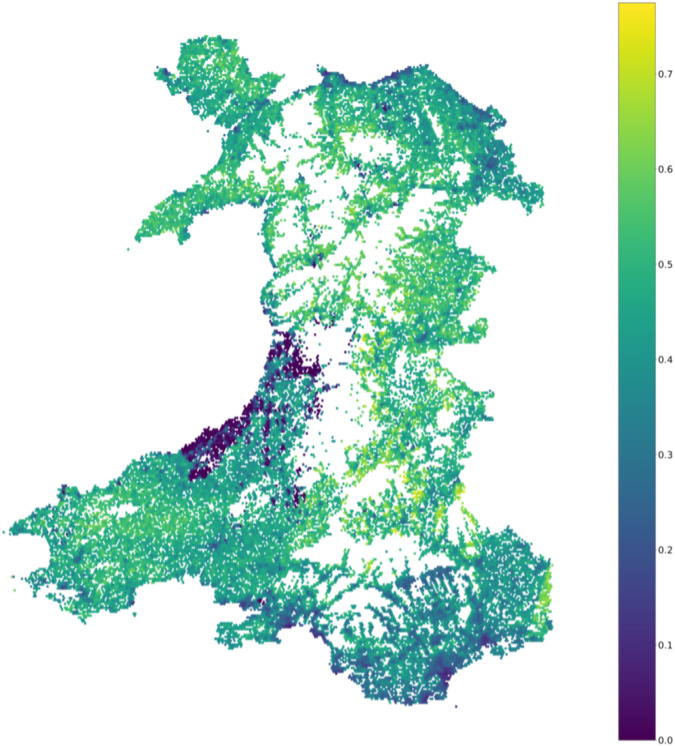
Mean EVI scores per household for Wales in 2018. Low EVI values are represented with purples and blues. Higher EVI values are represented with greens and yellows. The total range of EVI is 0–1.

The average EVI for 300 m buffers around residential addresses in Wales was 0.28 (Table 1). This falls within the healthy-vegetation range for EVI (0.2–0.8). The spatial distribution of household greenness across Wales is consistent with the theoretical principles of an EVI estimate, with rural areas having higher EVI values than urban regions. Figure 3 shows the distribution of greenness for households. Areas where there are no households to are shown in white. Figure 3 shows rural areas have higher average EVI scores than those found in coastal and more populated areas. The northwest, southwest and mid-Wales have the highest greenness scores. These are the most rural regions of the country and there are several managed green spaces, including national parks, forests found in these areas. Residential locations in south Wales cities and coastal towns consistently had the lowest EVI values across the study period.

### Associations between EVI values and green space type

Table 2 shows that for a nation-scale model, when total green space area is 0, EVI is predicted to be 0.24. This is comparable with the mean EVI of 0.28 reported in Table 1. The Pearson correlation coefficient is 0.33 and the adjusted r^2^ highlights that total green space area accounts for 11% of the variation in EVI for Wales. The difference between urban and rural mean EVI are described by the intercept values of 0.25 for urban areas and 0.34 for rural areas. In urban areas, the Pearson correlation coefficient is 0.57 and the adjusted r^2^ highlights that total green space area accounts for 32% of variation in EVI in urban areas. In rural areas, the Pearson correlation coefficient is 0.26 and the adjusted r^2^ highlights that average garden size can account for 7% of the variation in EVI in rural areas.

**Table 2:** Regression coefficients for Enhanced Vegetation Index and total green space area (m2) for Wales and stratified by urban/rural status.

	*Coefficient*	*95% CI*	*P value*	*R*	*Adjusted r2*
**Wales**				0.33	0.11
* Intercept*	0.2444	(0.244, 0.245)	< 0.001		
* Total green space area (m2)*	0.0000	(0.000, 0.000)	< 0.001		
**Urban**				0.57	0.32
* Intercept*	0.1878	(0.188, 0.188)	< 0.001		
* Total green space area (m2)*	0.0000	(0.000, 0.000)	< 0.001		
**Rural**				0.26	0.07
* Intercept*	0.3346	(0.334, 0.335)	< 0.001		
* Total green space area (m2)*	0.0000	(0.000, 0.000)	< 0.001		

Table 4 shows that when average garden size is 0, the model predicts that EVI will be 0.25 (mean EVI = 0.28, Fig. 1). The Wales-wide model predicts that when average garden size increases by 1 m^2^, EVI increases by 0.0001. Conversely, to see a 0.1 unit increase in EVI index, garden size would need to increase by 100 m^2^ (average garden size = 275 m^2^, Table 1). The Pearson correlation coefficient is 0.45 and the adjusted r^2^ highlights that average garden size can account for 20% of the variation in EVI. This means that 80% of the variation in EVI cannot be explained by average garden size alone. The difference between urban and rural mean EVI are described by the intercept values of 0.21 for urban areas and 0.33 for rural areas. In urban areas, as average garden size increases by 1 m^2^, EVI increases by 0.0002. Therefore, in urban areas, to see a 0.1 unit increase in EVI index score, garden size would need to increase by 50 m^2^. The Pearson correlation coefficient is 0.40 and the adjusted r^2^ highlights that averge garden size accounts for 16% of variation in EVI in urban areas. In rural areas, average garden size does not contribute to a measurable increase in EVI (β = 0.0000, 95% CI: 0.0000, 0.0000). The Pearson correlation coefficient is 0.34 and the adjusted r^2^ highlights that average garden size can account for 11% of the variation in EVI in rural areas. The very small β values in Tables 3 and 4 suggest no measurable, real world relationship.

**Table 3:** Regression coefficients for Enhanced Vegetation Index and average garden size (m2) for Wales and stratified by urban/rural status.

	*Coefficient*	*95% CI*	*P value*	*R*	*Adjusted r2*
**Wales**				0.45	0.20
* Intercept*	0.2493	(0.249, 0.250)	< 0.001		
* Average garden size (m2)*	0.0001	(0.000, 0.000)	< 0.001		
**Urban**				0.40	0.16
* Intercept*	0.2060	(0.206, 0.206)	< 0.001		
* Average garden size (m2)*	0.0002	(0.000, 0.000)	< 0.001		
**Rural**				0.34	0.11
* Intercept*	0.3311	(0.331, 0.331)	< 0.001		
* Average garden size (m2)*	0.0000	(0.000, 0.000)	< 0.001

**Table 4:** *Pearson correlation coefficient for Enhanced Vegetation Index and green space types with 95% confidence intervals*.

Green space type	Pearson Correlation	95% CI
*Meadow*	0.21	(0.18, 0.25)
*Park*	0.13	(0.12, 0.14)
*Allotments*	0.07	(0.06, 0.07)
*Quarry*	0.07	(0.07, 0.08)
*Religious grounds (e.g. cemeteries)*	0.06	(0.05. 0.06)
*Botanical gardens*	0.06	(0.04, 0.08)
*Play areas*	0.04	(0.03, 0.04)
*School grounds*	0.02	(0.02, 0.02)
*Mixed*	0.02	(0.02, 0.02)
*Sports pitches*	0.02	(0.01, 0.02)
*Other grounds*	0.01	(0.01, 0.01)
*Recreational*	0.01	(0.01, 0.02)
*Moor /heath*	0.00	(0.00, 0.00)
*Coniferous woodland*	−0.01	(−0.01, −0.01)
*Deciduous woodland*	−0.01	(−0.02, −0.01)
*Grassland*	−0.01	(−0.01, −0.01)
*Marsh*	−0.01	(−0.01, −0.01)

When stratifying total publicly accessible green space by type, we observed no moderate or strong positive associations with EVI using Pearson correlation (Table 4).

## Discussion

In this paper, we calculated three exposure assessment measures for a national population located across rural and urban regions. We compared a satellite-derived greenness exposure measure (EVI) with two vector-based measures of access to public and private green space. Our results indicated that satellite-derived measures such as EVI offer the opportunity to measure exposure to greenness for populations across large spatial and temporal scales in an objective and uniform way. EVI quantifies vegetation greenness and is an indicator of biomass [63] therefore, greater EVI values may indicate more vegetation by area *and*/*or* by volume (i.e. a greater EVI value does not necessarily equal a larger green space by area). Therefore, care should be taken when interpreting defined incremental changes of EVI (e.g. 0.1 or interquartile range) within a 300 m buffer zone as it is not possible to translate what incremental changes in EVI represent beyond changes in overall greenness. Our results are generalisable for temperate climates in the Northern Hemisphere.

Our work supports recent findings where a measure of access to green space was weakly correlated with NDVI [64]. Our results also support previous research findings that satellite-derived measures may be the most efficient way to measure population-wide, longitudinal exposures. Increases in both greenness and access to green space are positively associated with health outcomes around the globe [65–68]. Previous findings have reported that a defined increment of EVI (e.g. 0.1 or interquartile range) within a 300 m buffer zone is associated with improvements in health outcomes [69]. However, these studies do not indicate how these incremental changes can be translated for policy and practice in how to specifically modify the built environment to provide health-promoting environments [47]. Beyond promoting general greening policy, current evidence is not able to articulate which modifiable aspects of the built environment should be promoted or invested in by planners and policy makers. Therefore, the results of this study are an important contribution in interpreting epidemiological evidence on the relationship between EVI and health outcomes.

Our results highlight that EVI values do not readily map on to planning and policy defined green space types because these green space types are generally not characterised by a single vegetation type. This highlights the challenge of translating vegetation indices into actionable recommendations for planners and policy makers. Given current data availability for longitudinal research, satellite data derived EVI measures have limited ability to identify hyperlocal variations ( < 300 m) in public green spaces where multiple facilities or features may be present within the vicinity (e.g., a park, roadside trees, or allotments). Our findings suggest that greenness and total greenspace are not linearly related and both measures should be acknowledged as distinct exposures. It is challenging to produce policy that improves complex public health issues when there is heterogeneity in the methods used to defining exposure to greenness and access to green space [7]. Distinguishing more clearly between greenness and access to greenspace will help researchers, policy makers and practitioners to better understand exposures and mechanisms that drive health outcomes. A further implication of this finding is that multi-exposure models should be implemented to better understand the cumulative impact of different aspects of nature on health outcomes (i.e. household greenness and local neighbourhood access to green spaces). A final implication for future research is that our study highlights the need to investigate more detailed features of green spaces including manmade features (e.g. footpaths, kiosks, toilets).

Estimating green space exposure using satellite imagery was challenging because Wales does not experience many cloud-free days; even fewer when considering the cycle of a satellite recording data. As such we adopted a flexible approach to estimating EVI (i.e., we used different Landsat sensors to enable EVI measures to be calculated throughout the study period). We acknowledge that finer-resolution satellite data may yield different results, but it was not possible to obtain cloud free images for the wider study period [45, 50] (2008–2019) with any other open source satellite data. We found that either the data were not recorded for the entire study period, or it was not possible to create an annual image of Wales with the data available. However, satellite schemes such as Sentinel [70] offer the potential for higher resolution data and are recorded more frequently from 2016. We also excluded land that was privately owned such as farmland. Although this rural land type potentially provides valuable opportunities for exposure (e.g. via views), we chose to reflect the potential to access a parcel of land. We acknowledge that ‘accessibility’ is in fact a much more complex construct dependent on multiple characteristics of spaces, individuals, communities, and transport/pedestrian networks. Our classification is necessarily pragmatic and restricted to the data available at a national scale. However, it allows a nuanced understanding of green spaces which can inform the protection, improvement, management, planning and funding of green and blues spaces. A final limitation to note was that the EVI buffers were Euclidean distances, and the access buffers were calculated from 300 m network distance. Although the buffers are not a like for like comparison in shape, at this scale, we are confident that this did not significantly impact our results. The buffers were appropriate representations of how individuals would engage with greenness (e.g., viewing green space in a straight line) and publicly and privately accessible green spaces (e.g., walking along a footpath to a park).

Our study explores associations within the hyperlocal environment (300 m). Further work should be undertaken to explore whether the relationships reported remain for other vegetation indices and commonly defined activity spaces e.g., 500 m, 800 m and 1600 m around the home environment. Future studies should also focus on qualities of green spaces and facilities within the green spaces to shed light on which modifiable aspects of green spaces should be focussed on by planners, to enable local planning authorities to consider design quality. More could be drawn from EVI as an indicator of biomass in future studies because areas of greater biomass tend to be associated with areas of greater biodiversity [71, 72]. This may prove particularly useful in providing evidence to support health policy as evidence suggests that biodiversity may support pathways linked with positive health outcomes [73].

Satellite-derived measures such as EVI offer the opportunity to calculate objective and uniformly measured exposures of exposure to green space. There are many advantages of satellite-derived green space exposures, and currently these are the only feasible option for studies investigating large spatial and temporal scales. However, differences between EVI values do not necessarily reflect greater or lesser access, or different types of publicly accessible green space, nor capture greenspace signatures in three dimensions. Our results suggest that when characterising the hyperlocal green space environment, exposure to greenness and access to green spaces are distinct features of the environments that we live, work and play in. When investigating the impact of exposure to green spaces on health outcomes, particularly understanding mechanisms that rely on *using* a green space, satellite-derived measures should be supplemented with alternative data sources such as administrative and crowd-sources data to characterise green space boundaries and the facilities within them.

## Supplementary Information

Supplementary material

## Data Availability

The data used in this study were generated and stored in the Geographic Information Science Secure e-Research Platform (GIS SeRP) research platform at Swansea University, Swansea, UK. All proposals to use data from the GIS SeRP are subject to review by an independent Information Governance Review Panel (IGRP). Information on the application process can be found at: https://www.saildatabank.com/application-process.

## References

[1] Gascon M, Mas MT, Martínez D, Dadvand P, Forns J, Plasència A, et al. Mental health benefits of long-term exposure to residential green and blue spaces: a systematic review. Int J Environ Res Public Health. 2015;12:4354–79. 25913182 10.3390/ijerph120404354 PMC4410252

[2] Gascon M, Zijlema W, Vert C, White MP, Nieuwenhuijsen MJ. Outdoor blue spaces, human health and well-being: a systematic review of quantitative studies. Int J Hyg Environ Health. 2017;220:1207–21. 10.1016/j.ijheh.2017.08.004. 28843736 10.1016/j.ijheh.2017.08.004

[3] Yuan Y, Huang F, Lin F, Zhu P, Zhu P. Green space exposure on mortality and cardiovascular outcomes in older adults: a systematic review and meta-analysis of observational studies. Aging Clin Exp Res. 2020;33:1783–97. https://link.springer.com/article/10.1007/s40520-020-01710-0. 32951189 10.1007/s40520-020-01710-0

[4] Markevych I, Schoierer J, Hartig T, Chudnovsky A, Hystad P, Dzhambov AM, et al. Exploring pathways linking greenspace to health: theoretical and methodological guidance. Environ Res. 2017;158:301–17. 10.1016/j.envres.2017.06.028. 28672128 10.1016/j.envres.2017.06.028

[5] Van Dillen SME, De Vries S, Groenewegen PP, Spreeuwenberg P. Greenspace in urban neighbourhoods and residents’ health: adding quality to quantity. J Epidemiol Community Health. 2012;66:1–5. 21715445 10.1136/jech.2009.104695

[6] Mitchell R, Popham F. Effect of exposure to natural environment on health inequalities: an observational population study. Lancet. 2008;372:1655–60. 10.1016/S0140-6736(08)61689-X. 18994663 10.1016/S0140-6736(08)61689-X

[7] Cronin-de-chavez A, Islam S, Mceachan RRC. Health & place not a level playing field: a qualitative study exploring structural, community and individual determinants of greenspace use amongst low-income multi-ethnic families. Health Place 2019;56:118–26. 10.1016/j.healthplace.2019.01.018. 30735881 10.1016/j.healthplace.2019.01.018

[8] Vanaken G-JJ, Danckaerts M. Impact of green space exposure on children’s and adolescents’ mental health: a systematic review. Int J Environ Res Public Health. 2018;15:2668 https://www.mdpi.com/1660-4601/15/12/2668/htm. 30486416 10.3390/ijerph15122668 PMC6313536

[9] Egorov AI, Mudu P, Braubach M, Martuzzi M. *Urban green spaces and health: a review of evidence [Internet]*. WHO Regional Office for Europe. 2016. Available from: http://www.euro.who.int/__data/assets/pdf_file/0005/321971/Urban-green-spaces-and-health-review-evidence.pdf?ua=1.

[10] Dadvand P, Wright J, Martinez D, Basagaña X, McEachan RRC, Cirach M, et al. Inequality, green spaces, and pregnant women: Roles of ethnicity and individual and neighbourhood socioeconomic status. Environ Int. 2014;71:101–8. 24997306 10.1016/j.envint.2014.06.010

[11] Dzhambov A, Hartig T, Markevych I, Tilov B, Dimitrova D. Urban residential greenspace and mental health in youth: Different approaches to testing multiple pathways yield different conclusions. Environ Res. 2018;160:47–59. 28961469 10.1016/j.envres.2017.09.015

[12] Sarkar C. Residential greenness and adiposity: findings from the UK Biobank. Environ Int. 2017;106:1–10. 10.1016/j.envint.2017.05.016. 28551493 10.1016/j.envint.2017.05.016

[13] Zare Sakhvidi MJ, Knobel P, Bauwelinck M, de Keijzer C, Boll LM, Spano G, et al. Greenspace exposure and children behavior: a systematic review. Sci Total Environ. 2022;824:153608. 35134416 10.1016/j.scitotenv.2022.153608

[14] Alcock I, White MP, Lovell R, Higgins SL, Osborne NJ, Husk K, et al. What accounts for “England’s green and pleasant land”? A panel data analysis of mental health and land cover types in rural England. Landsc Urban Plan. 2015;142:38–46. 10.1016/j.landurbplan.2015.05.008.

[15] Dennis M, James P. Evaluating the relative influence on population health of domestic gardens and green space along a rural-urban gradient. Landsc Urban Plan. 2017;157:343–51. 10.1016/j.landurbplan.2016.08.009.

[16] Hughey SM, Kaczynski AT, Child S, Moore JB, Porter D, Hibbert J. Green and lean: is neighborhood park and playground availability associated with youth obesity? Variations by gender, socioeconomic status, and race / ethnicity. Prev Med. 2017;95:101–8. 10.1016/j.ypmed.2016.11.024. 10.1016/j.ypmed.2016.11.024 27932053

[17] Haklay M, Weber P. OpenStreet map: User-generated street maps. IEEE Pervasive Comput. 2008;7(4):12–8.

[18] Dimitrova DD, Dzhambov AM. Perceived access to recreational/green areas as an effect modifier of the relationship between health and neighbourhood noise/air quality: results from the 3rd European Quality of Life Survey (EQLS, 2011–2012). Urban Urban Green. 2017;23:54–60.

[19] Sulander T, Karvinen E, Holopainen M. Urban green space visits and mortality among older adults. Epidemiology. 2016;27:e34–5. https://journals.lww.com/epidem/Fulltext/2016/09000/Urban_Green_Space_Visits_and_Mortality_Among_Older.26.aspx. 27327021 10.1097/EDE.0000000000000511

[20] Wilkins EL, Morris MA, Radley D, Griffiths C. Using Geographic Information Systems to measure retail food environments: Discussion of methodological considerations and a proposed reporting checklist (Geo-FERN). Heal Place. 2017;44:110–7. 10.1016/j.healthplace.2017.01.008 28236788

[21] Labib SM, Lindley S, Huck JJ. Spatial dimensions of the influence of urban green-blue spaces on human health: a systematic review. Environ Res. 2020;180:108869. 31722804 10.1016/j.envres.2019.108869

[22] Young NE, Anderson RS, Chignell SM, Vorster AG, Lawrence R, Evangelista PH. A survival guide to Landsat preprocessing. Ecology. 2017;98:920–32. 28072449 10.1002/ecy.1730

[23] Wulder MA, Coops NC, Roy DP, White JC, Hermosilla T. Land cover 2.0. Int J Remote Sens. 2018;39:4254–84.

[24] USGS. *EarthExplorer*. 2020. Available from: https://earthexplorer.usgs.gov/.

[25] Rugel EJ, Henderson SB, Carpiano RM, Brauer M. Beyond the Normalized Difference Vegetation Index (NDVI): developing a natural space index for population-level health research. Environ Res. 2017;159:474–83. 28863302 10.1016/j.envres.2017.08.033

[26] Dare PM. Shadow analysis in high-resolution satellite imagery of urban areas. 2005.

[27] Su JG, Dadvand P, Nieuwenhuijsen MJ, Bartoll X, Jerrett M. Associations of green space metrics with health and behavior outcomes at different buffer sizes and remote sensing sensor resolutions. Environ Int. 2019;126:162–70. 30798197 10.1016/j.envint.2019.02.008

[28] Small C. Estimation of urban vegetation abundance by spectral mixture analysis. 2001;22:1305–34. 10.1080/01431160151144369.

[29] James P, Banay RF, Hart JE, Laden F. A review of the health benefits of greenness. Curr Epidemiol Rep. 2015;2:131–42. http://link.springer.com/10.1007/s40471-015-0043-7. 26185745 10.1007/s40471-015-0043-7 PMC4500194

[30] de Keijzer C, Foraster M, Basagaña X, Tonne C, Garcia LA, Valentín A, et al. Long-term greenspace exposure and progression of arterial stiffness: the Whitehall ii cohort study. Environ Health Perspect. 2020;128:1–9. 10.1289/EHP6159. 10.1289/EHP6159 PMC7319656 32589457

[31] Orioli R, Antonucci C, Scortichini M, Cerza F, Marando F, Ancona C, et al. Exposure to residential greenness as a predictor of cause-specific mortality and stroke incidence in the Rome longitudinal study. Environ Health Perspect. 2019;127:27002. https://ehp.niehs.nih.gov/doi/full/10.1289/EHP2854. 30775931 10.1289/EHP2854 PMC6752936

[32] Rugel EJ, Carpiano RM, Henderson SB, Brauer M. Exposure to natural space, sense of community belonging, and adverse mental health outcomes across an urban region. Environ Res. 2019;171:365–77. 10.1016/j.envres.2019.01.034. 30716514 10.1016/j.envres.2019.01.034

[33] Yang BY, Markevych I, Heinrich J, Bowatte G, Bloom MS, Guo Y, et al. Associations of greenness with diabetes mellitus and glucose-homeostasis markers: the 33 Communities Chinese Health Study. Int J Hyg Environ Health. 2019;222:283–90. 30545606 10.1016/j.ijheh.2018.12.001

[34] Brown SC, Perrino T, Lombard J, Wang K, Toro M, Rundek T, et al. Health disparities in the relationship of neighborhood greenness to mental health outcomes in 249,405 U.S. medicare beneficiaries. Int J Environ Res Public Heal. 2018;15:430. https://www.mdpi.com/1660-4601/15/3/430/htm. 10.3390/ijerph15030430 PMC5876975 29494513

[35] Pereira G, Foster S, Martin K, Christian H, Boruff BJ, Knuiman M, et al. The association between neighborhood greenness and cardiovascular disease: an observational study. BMC Public Health. 2012;12:1–9. https://bmcpublichealth.biomedcentral.com/articles/10.1186/1471-2458-12-466. 22720780 10.1186/1471-2458-12-466 PMC3476430

[36] Lane KJ, Stokes EC, Seto KC, Thanikachalam S, Thanikachalam M, Bell ML. Associations between greenness, impervious surface area, and nighttime lights on biomarkers of vascular aging in Chennai, India. Environ Health Perspect. 2017;125. 10.1289/EHP541. 10.1289/EHP541 PMC5783666 28886599

[37] Slawsky ED, Hajat A, Rhew IC, Russette H, Semmens EO, Kaufman JD, et al. Neighborhood greenspace exposure as a protective factor in dementia risk among U.S. adults 75 years or older: a cohort study. Environ Heal A Glob Access Sci Source. 2022;21:1–10. https://ehjournal.biomedcentral.com/articles/10.1186/s12940-022-00830-6. 10.1186/s12940-022-00830-6 PMC8760791 35033073

[38] Dadvand P, Bartoll X, Basagaña X, Dalmau-Bueno A, Martinez D, Ambros A, et al. Green spaces and general health: roles of mental health status, social support, and physical activity. Environ Int. 2016;91:161–7. 10.1016/j.envint.2016.02.029. 26949869 10.1016/j.envint.2016.02.029

[39] Houlden V, Weich S, de Albuquerque JP, Jarvis S, Rees K. The relationship between greenspace and the mental wellbeing of adults: a systematic review. PLoS ONE. 2018;13:1–35. 10.1371/journal.pone.0203000 PMC6135392 30208073

[40] Paul LA, Hystad P, Burnett RT, Kwong JC, Crouse DL, van Donkelaar A, et al. Urban green space and the risks of dementia and stroke. Environ Res. 2020;186:109520. 32344208 10.1016/j.envres.2020.109520

[41] Barboza EP, Cirach M, Khomenko S, Iungman T, Mueller N, Barrera-Gómez J. et al. Green space and mortality in European cities: a health impact assessment study. Lancet Planet Heal. 2021;5:e718–e30. 10.1016/S2542-5196(21)00229-1 34627476

[42] Twohig-Bennett C, Jones A. The health benefits of the great outdoors: a systematic review and meta-analysis of greenspace exposure and health outcomes. Environ Res. 2018;166:628–37. 29982151 10.1016/j.envres.2018.06.030 PMC6562165

[43] Gitelson AA. Wide dynamic range vegetation index for remote quantification of biophysical characteristics of vegetation. J Plant Physiol. 2004;161:165–73. 15022830 10.1078/0176-1617-01176

[44] Klompmaker JO, Hoek G, Bloemsma LD, Gehring U, Strak M, Wijga AH, et al. Green space definition affects associations of green space with overweight and physical activity. Environ Res. 2018;160:531–40. 10.1016/j.envres.2017.10.027. 29106952 10.1016/j.envres.2017.10.027

[45] Thompson DA, Geary RS, Rowney FM, Fry R, Watkins A, Wheeler BW, et al. Cohort profile: the green and blue spaces (GBS) and mental health in Wales e-cohort. Int J Epidemiol. 2022;51:e285–94. 10.1093/ije/dyac080 PMC9558062 35446420

[46] Sadeh M, Brauer M, Dankner R, Fulman N, Chudnovsky A. Remote sensing metrics to assess exposure to residential greenness in epidemiological studies: a population case study from the Eastern Mediterranean. Environ Int. 2021;146:106270. 33276312 10.1016/j.envint.2020.106270

[47] Martinez AdelaI, Labib SM. Demystifying normalized difference vegetation index (NDVI) for greenness exposure assessments and policy interventions in urban greening. Environ Res. 2023;220:115155 10.1016/j.envres.2022.115155. 36584843 10.1016/j.envres.2022.115155

[48] Huete A, Justice C, Liu H. Development of vegetation and soil indices for MODIS-EOS. Remote Sens Environ. 1994;49:224–34.

[49] Huete AR, Liu HQ, Batchily K, Van Leeuwen W. A comparison of vegetation indices over a global set of TM images for EOS-MODIS. Remote Sens Environ. 1997;59:440–51.

[50] Mizen A, Song J, Fry R, Akbari A, Berridge D, Parker SC, et al. Longitudinal access and exposure to green-blue spaces and individual-level mental health and well-being: protocol for a longitudinal, population-wide record-linked natural experiment. BMJ Open. 2019;9:1–10. 10.1136/bmjopen-2018-027289. 10.1136/bmjopen-2018-027289 PMC6528002 31005938

[51] Garrett JK, Rowney FM, White MP, Lovell R, Fry RJ, Akbari A, et al. Visiting nature is associated with lower socioeconomic inequalities in well-being in Wales. Sci Rep. 2023;13:1–13. 10.1038/s41598-023-35427-7. 37322030 10.1038/s41598-023-35427-7 PMC10272170

[52] World Health Organization. Urban green spaces: a brief for action. Reg Off Eur. 2017;24. http://www.euro.who.int/__data/assets/pdf_file/0010/342289/Urban-Green-Spaces_EN_WHO_web.pdf?ua=1.

[53] Beck HE, Zimmermann NE, McVicar TR, Vergopolan N, Berg A, Wood EF. Present and future Köppen-Geiger climate classification maps at 1-km resolution. Sci Data. 2018;5:1–12. https://www.nature.com/articles/sdata2018214. 30375988 10.1038/sdata.2018.214 PMC6207062

[54] Mohanasundaram S, Baghel T, Thakur V, Udmale P, Shrestha S. Reconstructing NDVI and land surface temperature for cloud cover pixels of Landsat-8 images for assessing vegetation health index in the Northeast region of Thailand. Environ Monit Assess. 2022;195:1–34. https://link.springer.com/article/10.1007/s10661-022-10802-5. 36534216 10.1007/s10661-022-10802-5

[55] QGIS. *QGIS Python Plugins Repository [Internet]*. 2020 [cited 2020 Apr 2]. Available from: https://plugins.qgis.org/plugins/SemiAutomaticClassificationPlugin/.

[56] QGIS. *Cloud Masking - Qgis plugin [Internet]*. 2020 [cited 2020 Apr 2]. Available from: https://plugins.qgis.org/plugins/CloudMasking/.

[57] *QGIS. i.vi - GRASS GIS manual [Internet]*. 2020 [cited 2020 Apr 2]. Available from: https://grass.osgeo.org/grass78/manuals/i.vi.html.

[58] Huete A, Didan K, Miura T, Rodriguez EP, Gao X, Ferreira LG. Overview of the radiometric and biophysical performance of the MODIS vegetation indices. Remote Sens Environ. 2002;83:195–213. https://www.elsevier.com/locate/rse.

[59] Ordnance Survey. *OS MasterMap Topography Layer*. 2017. Available from: https://www.ordnancesurvey.co.uk/business-and-government/products/topography-layer.html.

[60] PostGIS. *PostGIS — Spatial and Geographic Objects for PostgreSQL*. 2017. Available from: http://postgis.net/.

[61] Ordnance Survey. *OS MasterMap Greenspace Layer | Greenspace Mapping Product*. 2020. Available from: https://www.ordnancesurvey.co.uk/business-government/products/mastermap-greenspace.

[62] ONS. *Rural and Urban Area Classification*. 2004. Available from: http://www.ons.gov.uk/ons/guide-method/geography/products/area-classifications/rural-urban-definition-and-la/rural-urban-definition--england-and-wales-/index.html.

[63] Lu D. *The potential and challenge of remote sensing‐based biomass estimation*. 2007;27:1297–328. 10.1080/01431160500486732.

[64] Larkin A, Hystad P. Evaluating street view exposure measures of visible green space for health research. J Expo Sci Environ Epidemiol. 2018;29(4):447–56. https://www.nature.com/articles/s41370-018-0017-1. 29352209 10.1038/s41370-018-0017-1

[65] Song H, Lane KJ, Kim H, Kim H, Byun G, Le M, et al. Association between urban greenness and depressive symptoms: evaluation of greenness using various indicators. Int J Environ Res Public Health. 2019;16(2):173. 30634488 10.3390/ijerph16020173 PMC6352234

[66] Yu L, Li T, Yang Z, Zhang X, Xu L, Wu Y, et al. Long-term exposure to residential surrounding greenness and incidence of diabetes: a prospective cohort study. Environ Pollut. 2022;310:119821 https://linkinghub.elsevier.com/retrieve/pii/S0269749122010351. 35870530 10.1016/j.envpol.2022.119821

[67] Helbich M, Klein N, Roberts H, Hagedoorn P, Groenewegen PP. More green space is related to less antidepressant prescription rates in the Netherlands: a Bayesian geoadditive quantile regression approach. Environ Res. 2018;166:290–7. 10.1016/j.envres.2018.06.010. 29936285 10.1016/j.envres.2018.06.010

[68] White MP, Elliott LR, Grellier J, Economou T, Bell S, Bratman GN, et al. Associations between green/blue spaces and mental health across 18 countries. Sci Rep. 2021;11:8903 10.1038/s41598-021-87675-0. 33903601 10.1038/s41598-021-87675-0 PMC8076244

[69] Min KD, Kim JS, Park YH, Shin HY, Kim C, Seo SW, et al. New assessment for residential greenness and the association with cortical thickness in cognitively healthy adults. Sci Total Environ. 2021;778:146129 10.1016/j.scitotenv.2021.146129. 33714817 10.1016/j.scitotenv.2021.146129

[70] Sentinel Online. *Sentinel Online - ESA - Sentinel Online [Internet]*. 2020. https://sentinels.copernicus.eu/web/sentinel/home.

[71] Cardinale BJ, Wright JP, Cadotte MW, Carroll IT, Hector A, Srivastava DS, et al. Impacts of plant diversity on biomass production increase through time because of species complementarity. Proc Natl Acad Sci USA. 2007;104:18123–8. https://www.pnas.org/doi/abs/10.1073/pnas.0709069104. 17991772 10.1073/pnas.0709069104 PMC2084307

[72] Aerts R, Honnay O, Van Nieuwenhuyse A. Biodiversity and human health: mechanisms and evidence of the positive health effects of diversity in nature and green spaces. British Medical Bulletin. 2018;127:5–22. 30007287 10.1093/bmb/ldy021

[73] Marselle MR, Hartig T, Cox DTC, de Bell S, Knapp S, Lindley S, et al. Pathways linking biodiversity to human health: a conceptual framework. Environ Int. 2021;150:106420. 33556912 10.1016/j.envint.2021.106420

