# Supplementary material for: The use of Enhanced Vegetation Index for assessing access to different types of green space in epidemiological studies
Source: J Expo Sci Environ Epidemiol. 2024 Feb 29;34(5):753–60. doi: 10.1038/s41370-024-00650-5 (PMC11446865; doi:10.1038/s41370-024-00650-5)
Supplement: Supplementary file 1 — Supplementary material [file 41370_2024_650_MOESM1_ESM.docx]

**Supplementary material**

Table A: Green and Blue Spaces Typology Tiers

| **Tier 1** | **Tier 2** | **Tier 3** | **Description** |
| --- | --- | --- | --- |
| Amenity | Recreation Space | Parks | Open to the public, areas of managed, open land. Often with grass and formal flowerbeds and/or trees |
|  |  | Recreation areas | A purpose-built area used for playing games and social activities e.g., bowling green, crazy golf, members golf club |
|  |  | Sports pitches | Formal, maintained sports pitches (may be Astroturf/3G) |
|  |  | Play areas | A public area with swings, slides, climbing frames, etc… |
|  | Private | Domestic gardens | A plot of ground adjacent to an addressed house |
| Functional | Productive | City farms | A farm within an urban area (often run educational programmes) |
|  |  | Allotments | Small allocation of land, normally rented from a Local Authority |
|  | Burial Grounds | Cemeteries | An area where the dead are buried |
|  |  | Churchyard | An area where the dead are buried, including graveyards and churchyards |
|  | Institutional grounds | School grounds | A defined area belonging to a school premises |
|  |  | Other grounds | An area of land that is associated with a property |
|  | Gardens | Botanical Gardens | An area of land containing plants of scientific interest and facilities for their care and cultivation |
|  |  | Community garden | A single piece of land gardened collectively by a group of people |
| Semi-natural habitat | Wetland | Marsh | An area of land that is waterlogged throughout the year |
|  | Woodland | Deciduous woodland | An area of ground covered with deciduous trees |
|  |  | Coniferous woodland | An area of ground covered with coniferous trees |
|  |  | Mixed woodland | An area of ground covered with diverse types of trees |
|  | Other Habitats | Moor/heath | An area of open, uncultivated, and usually high land with poor soil that is covered mainly with grass, heather, bilberry and sedges with very few trees or bushes |
|  |  | Grassland | Areas of bracken and uncultivated grassland, often tufted with a 'hummocky' appearance, and normally found on the higher parts of hills, mountains, and down land |
|  |  | Quarry | An area that has been dug for the purpose of extracting rock that is no longer active |
|  |  | Meadow | An area of fertile grassland on the flood plain of a river |
| Linear | Inland | River | Water flowing in a definite channel towards the sea, a lake or into another river |
|  |  | Canal | An artificial watercourse for inland navigation |
|  |  | Transport corridors | A purpose-built route for taking people from one place to another (road, rail, cycleways and walking) |
|  | Coastal | Cliffs | A steep rock face, especially at the edge of the sea |
|  |  | Beach | An area of sand, shingles or rocks beside inland or tidal water |
|  |  | Marina | A small harbour or area of water where yachts and other pleasure craft can moor |
|  |  | Docklands | An area in a harbour where ships are loaded/unloaded or repaired |
|  |  | Estuary | The tidal mouth of a river where the channel broadens out at the coast |
|  |  | Harbour | An area of naturally or artificially protected water on a coast where boats can anchor or moor |
| Enclosed blue feature | Inland | Lake | A large area of salt or fresh water which may be surrounded by land and is larger than a pond |
|  |  | Reservoir | A natural or artificial lake or pond used for storing water which may be used for irrigation, water supply, hydro-electric power generation or flood control |
|  |  | Pond | A small area of fresh water, often artificially created, which is smaller than a lake |
|  |  | Outdoor Swimming Pool | Structure which contains water for swimming in |
|  | Coastal | Lido | A public open-air swimming pool or bathing beach |


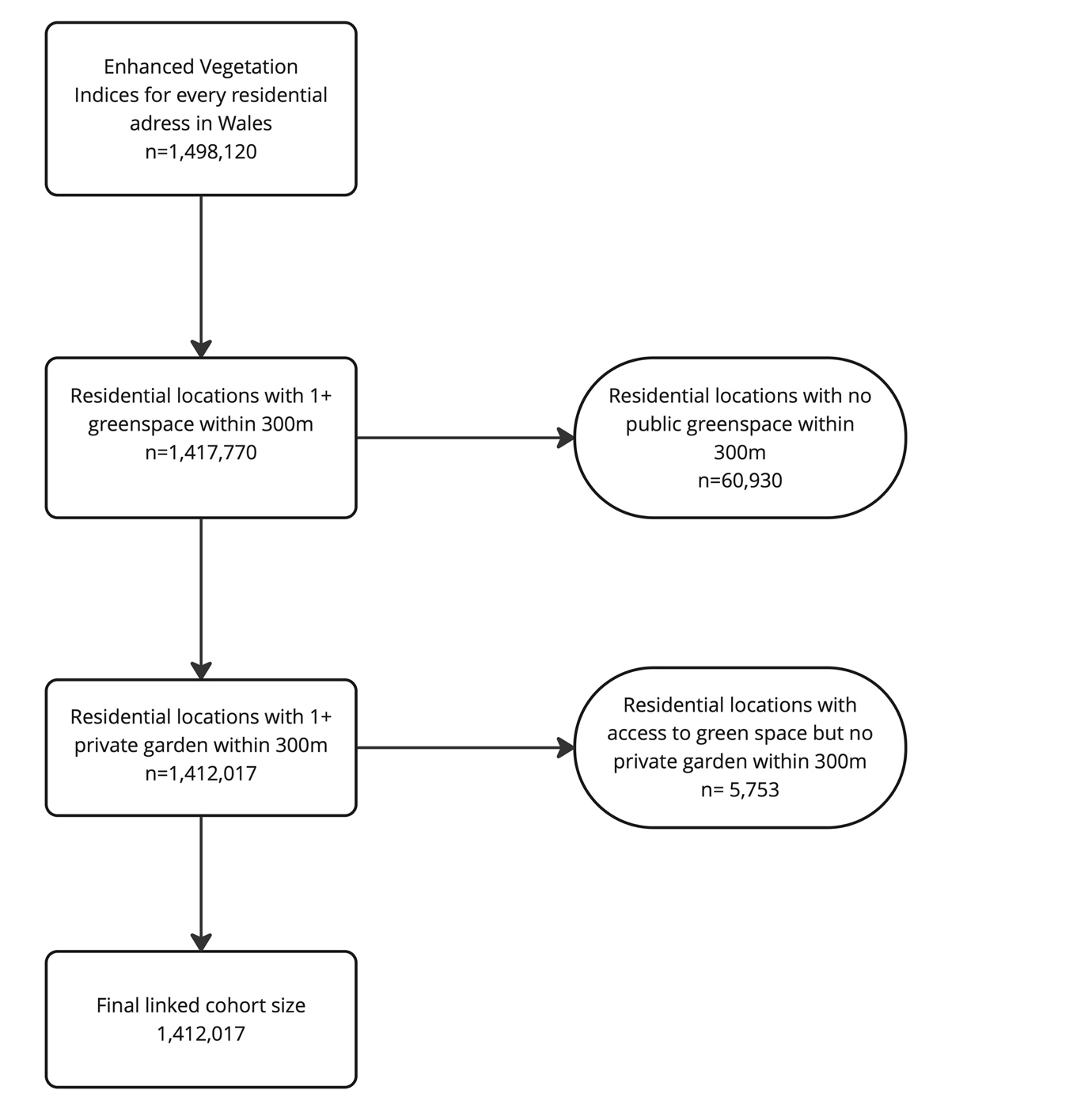


Figure A: Consort diagram of exposure datasets
